# Supplementary material for: Prevalence of diabetic retinopathy in children and young people living with diabetes: protocol for a systematic review
Source: BMJ Open. 2017 Nov 15;7(11):e018578. doi: 10.1136/bmjopen-2017-018578 (PMC5695488; doi:10.1136/bmjopen-2017-018578)
Supplement: Supplementary file 1 [file bmjopen-2017-018578supp001.pdf]

Additional file 2: Quality assessment

External validity

1. **Was the sampling frame a true or close representation of the target population?**

Yes: All patients registered in paediatric diabetes units or clinics, i.e. general population of children and young people living with diabetes.

No: Participants selected only among screening attenders, voluntary participants of diabetes summer camps or other events.

2. Are the study subjects described in detail regarding?

Yes: Age or diabetes duration was reported along with a measure of diabetes control and the year(s) when the study was conducted.

No: One or more of the previous details was not described.

3. Was some form of consecutive, random selection used to select the sample, OR was a census undertaken?

Yes: Yes.

No: No or unclear.

4. Was the likelihood of nonresponse bias minimal?

Yes: Response >50%.

No: Unclear, not reported, or less 50%.

Internal validity

5. Were data collected directly from the subjects (as opposed to a proxy)?

Yes: Cross-sectional and prospective studies, retrospective analysis of prospective databases e.g. diabetic retinopathy screening registers.

No: Retrospective analysis of medical records.

6. Was an acceptable case definition used in the study?

Yes: Internationally acceptable (DCCT compliant) taxonomy of diabetic retinopathy was used.

No: No clear definition of diabetic retinopathy, e.g. *retinal changes,* or the severity of cases was not described.

7. Was the study instrument that measured the parameter of interest shown to have validity and reliability?

Yes: Retinal photographs or fluorescein angiogram.

No: Fundoscopy or ophthalmoscopy alone.

8. Was the same mode of data collection used for all subjects?

Yes: all participants received the same diagnostic test including screening tests.

No: Triage tests were used or unclear.

9. Was the length of the shortest prevalence period for the parameter of interest appropriate?

Yes: all studies were considered adequate. This is a crucial question in the prevalence of diabetic retinopathy in children and young people.

10. Were the numerator(s) and denominator(s) for the parameter of interest appropriate and confidence interval given?

Yes: Confidence interval given.

No: Confidence interval not given.

11. Was the sample size equal or greater than 200?

Yes.

No.
