# Supplementary material for: Prevalence of diabetic retinopathy in children and young people living with diabetes: protocol for a systematic review
Source: BMJ Open. 2017 Nov 15;7(11):e018578. doi: 10.1136/bmjopen-2017-018578 (PMC5695488; doi:10.1136/bmjopen-2017-018578)
Supplement: Author's manuscript [file bmjopen-2017-018578.draft_revisions.pdf]

BMJ Open is committed to open peer review. As part of this commitment we make the peer review history of every article we publish publicly available.

When an article is published we post the peer reviewers' comments and the authors' responses online. We also post the versions of the paper that were used during peer review. These are the versions that the peer review comments apply to.

The versions of the paper that follow are the versions that were submitted during the peer review process. They are not the versions of record or the final published versions. They should not be cited or distributed as the published version of this manuscript.

BMJ Open is an open access journal and the full, final, typeset and author-corrected version of record of the manuscript is available on our site with no access controls, subscription charges or pay-per-view fees (http://bmjopen.bmj.com).

If you have any questions on BMJ Open's open peer review process please email editorial.bmjopen@bmj.com

# Prevalence of diabetic retinopathy in children and young people living with diabetes: protocol for a systematic review.

| | |
|---|---|
| Journal: | *BMJ Open* |
| Manuscript ID | bmjopen-2017-018578 |
| Article Type: | Protocol |
| Date Submitted by the Author: | 07-Jul-2017 |
| Complete List of Authors: | Ibanez-Bruron, Maria; UCL, GOS Institute of Child Health; UCL, Ulverscroft Vision Research Group<br>Solebo, AL; UCL, GOS Institute of Child Health; UCL, Ulverscroft Vision Research Group<br>Cumberland, Phillippa ; UCL, GOS Institute of Child Health; UCL, Ulverscroft Vision Research Group<br>Rahi, Jugnoo; UCL, GOS Institute of Child Health; UCL, Ulverscroft Vision Research Group |
| <b>Primary Subject Heading</b>: | Ophthalmology |
| Secondary Subject Heading: | Paediatrics, Diabetes and endocrinology, Epidemiology, Evidence based practice |
| Keywords: | Diabetic retinopathy < DIABETES & ENDOCRINOLOGY, EPIDEMIOLOGY, Paediatric ophthalmology < OPHTHALMOLOGY, PREVENTIVE MEDICINE |
| | |

SCHOLARONE™
Manuscripts

For peer review only

Title page

**Title:**

# Prevalence of diabetic retinopathy in children and young people living with diabetes: protocol for a systematic review.

**Authors**:
Maria C. Ibanez-Bruron MD; Ameenat L. Solebo FRCOphth; Phillippa M. Cumberland MSc; Jugnoo S. Rahi FRCOphth.

**Institutional address:**
GOS Institute of Child Health UCL, 30 Guildford Street, London, WC1N 1EH.

**Email addresses:**
m.bruron@ucl.ac.uk; a.solebo@ucl.ac.uk; p.cumberland@ucl.ac.uk; j.rahi@ucl.ac.uk

**Affiliations of authors:**
GOS Institute of Child Health, University College London, London, UK (MCI-B, ALS, PMC, JSR); Ulverscroft Vision Research Group, London, UK (MCI-B, ALS, PMC, JSR); Great Ormond Street Hospital for Children NHS Foundation Trust, London, UK (ALS, JSR); Moorfields Eye Hospital NHS Foundation Trust, London, UK (ALS, JSR); Institute of Ophthalmology, University College London, London, UK (ALS, JSR).

**Corresponding author:**
Professor Jugnoo Sangeeta Rahi FRCOphth.
GOS Institute of Child Health UCL, 30 Guildford Street, London, WC1N 1EH.
Tel: 020 7905 2250; email: j.rahi@ucl.ac.uk

For peer review only

## Abstract

**Introduction:** The frequency of diabetes mellitus in childhood is increasing. Thus, more children and young people are at risk of developing diabetic retinopathy and diabetes related visual impairment. However, there is no consensus on optimal screening strategies for the paediatric population reflecting the lack of clarity about the current burden of disease in this group. We aim to estimate the prevalence of diabetic retinopathy in children and young people living with types 1 or 2 diabetes, and to investigate potential sources of heterogeneity in this figure so as to inform screening strategies for this population.

**Methods and analysis:** PubMed and EMBASE will be searched from 1995 to 2016 using the OvidSP platform with no language restriction. Additionally, manual review of the references lists of included articles will be conducted. Two investigators will independently screen titles and abstracts for potential eligibility. Studies which report prevalence of diabetic retinopathy amongst general populations of children and young people with types 1 or 2 diabetes will be included. Pooled prevalence estimates of diabetic retinopathy reported in studies with sample size greater than 200 participants will be calculated by random effect model. Forest plots will be used to summarize individual and pooled estimates of the prevalence. Heterogeneity between studies will be assessed using the $I^2$ statistic and explored through meta-regressions and sub-group analyses if the necessary data are available.

**Ethics and dissemination:** Ethics approval is not required as this is a review of anonymised published data. We will report the findings of this systematic review in a peer-reviewed journal, and share it with the relevant professionals including health authorities through our DECS collaborative network.

For peer review only - http://bmjopen.bmj.com/site/about/guidelines.xhtml

**Systematic review registration:** PROSPERO (CRD42017067178).

**Strengths and limitations of this study**

- This formal systematic review will provide currently unavailable information on the prevalence of diabetic retinopathy in the paediatric population.
- Only studies reporting diagnostic methods and grades of diabetic retinopathy will be included to enhance precision.
- To increase the applicability of the findings, two severity cut-offs will be used to calculate prevalence estimates.
- As with all systematic reviews, the poor reporting of methods in primary studies might compromise the exploration of the heterogeneity.

For peer review only

## Background

Diabetic retinopathy is the most common preventable cause of visual impairment in young adults. [1] The rising prevalence of childhood type 1 and type 2 diabetes, [2-4] has led to a significant increase in the number of children and young people at risk of visual loss.

Microvascular retinal damage is a marker of general vascular health, indicative of a need for greater diabetic disease control. The more advanced, but still asymptomatic, stages of diabetic retinopathy require additional ophthalmic intervention to reduce the risk of irreversible visual loss. Screening for diabetic retinopathy in children and young people is recommended by many national population health programmes, with commencement of annual retinal examination determined either by age (typically age 10 – 12 years) or duration of disease (typically 2-5 years from diagnosis). [5-7]

Although it has been suggested that these recommendations need to be revised based on recent evidence on the burden of the disease in this population, there is no consensus on the direction of these modifications. [8-12] Variable estimates of the prevalence of diabetic retinopathy may be due in part to heterogeneity between study populations, diagnostic methods, or definitions of diabetic retinopathy used in the studies. [13-14] Consensus about screening strategies for the paediatric population required greater clarity in evidence base regarding the natural history and the burden of disease in this group. [15-17]

The aim of this systematic review is to estimate the prevalence of diabetic retinopathy in children and young people living with type 1 or type 2 diabetes, and to investigate potential sources of heterogeneity such as study sample characteristics

For peer review only

(e.g. age, diabetes type and duration, and glycaemic control), diagnostic methods and definition of diabetic retinopathy to inform screening strategies for the paediatric population.

**Methods/Design**

The systematic review will be performed in accordance with the Preferred Reporting Items for Systematic Reviews and Meta-Analyses (PRISMA) guidelines, with this protocol written according to the PRISMA-P checklist. [18] The PRISMA-P checklist can be found as Additional file 1. The study has been registered on PROSPERO (CRD42017067178).

**Data sources and searches**

The PubMed and EMBASE databases will be searched using the OvidSP platform. We will also manually review the references lists of identified articles. The search strategy (table 1) will include terms related to children or young people, diabetes, diabetic retinopathy, prevalence and diagnostic tests, and there will be no language restrictions. We will exclude studies published before January 1995 as older studies are likely to have been conducted prior to the publication of the Diabetes Control and Complications Trial (DCCT), which demonstrated decreased risk of diabetic retinopathy development or progression with tighter glycaemic control than that previously used in routine clinical practice, and may have led to significant changes in disease management and natural history. [19]

For peer review only

**Study eligibility criteria**

We will include studies which report prevalence estimates of diabetic retinopathy amongst general populations of children and young people with diabetes mellitus, or studies from which the prevalence data may be derived (table 2). Eligible studies will be those which meet the following criteria:

- Participants

Individuals aged under 18 years living with types 1 or 2 diabetes. Studies with median/mean age of the sample greater than 25 years or which include participants older than 30 years will be excluded.

- Disease measure

Report of the clinical examination method used to determine diabetic retinopathy status, and description of either retinal findings or grade of retinopathy using the Early Treatment Diabetic Retinopathy Study (ETDRS) taxonomy or equivalent. Prevalence will be calculated using two cut-offs, *any* diabetic retinopathy and *sight-threatening* diabetic retinopathy i.e. grades of retinopathy that require prompt ophthalmic management to reduce the risk of irreversible visual impairment. *Any* diabetic retinopathy will be defined as background retinopathy or more severe disease. *Sight-threatening* diabetic retinopathy [20] will be defined in this review as proliferative diabetic retinopathy or *clinically significant* maculopathy, i.e. any degree of oedema threatening the centre of the retina (the macula). Conference abstracts with sufficient data on disease measure to determine eligibility will be included.

For peer review only

**Study selection**

The search results will be extracted and managed using Microsoft Excel (Microsoft Corporation, Redmond, WA, USA). Two independent investigators (MIB, ALS) will screen titles and abstracts, and then apply the eligibility criteria to full-text articles. Disagreements will be resolved by consensus following discussion. Arbitration by a third author (JSR) will be sought whenever necessary.

**Data extraction**

Two investigators (MIB, ALS) will extract the data using a standardised form. The following data items will be extracted: first author, year of publication, design, country, year of study conduction, sample size, diabetes type, age at examination, diabetes duration, HbA1c, diagnostic test, diabetic retinopathy definition, number of any diabetic retinopathy cases, severity of diabetic retinopathy cases. If a study reports multiple prevalence estimates over time, the first prevalence estimate will be selected. Characteristics of children and young people with sight-threatening diabetic retinopathy will be also extracted. Authors will be contacted if data in the original publication is unclear.

**Assessment of risk of bias in included studies**

The risk of bias will be assessed with an adapted version of the tool developed by Hoy and colleagues [21] (additional file 2). The sample will be considered representative of the target population (i.e. ‘general’ population) when all children and young people registered in a paediatric diabetes clinic, or living in a specified catchment area were potentially eligible for the study. Sources of information will be classified as appropriate when diabetic retinopathy status was assessed during the

For peer review only

study (cross-sectional and prospective studies) or when prospective databases such as diabetic retinopathy screening registers were used.

**Data synthesis**

A comprehensive table for summary of findings with narrative description of all eligible studies will be reported. However, only studies with a sample size equal or greater than 200 participants will be included in the meta-analysis. Small studies will be excluded to reduce variability due to imprecise estimates. Pooled prevalence estimates of any diabetic retinopathy and of sight-threatening diabetic retinopathy will be calculated by random effect model meta-analysis. Forest plots will be used to summarize individual and pooled estimates of the prevalence. Heterogeneity between studies will be assessed using the $I^2$ statistic (values of 25%, 50% and 75% will be considered low, medium and high heterogeneity, respectively) [22] and will be further explored through meta-regressions and sub-group analyses considering the following covariates: sample median/mean age, median/mean diabetes duration, type of diabetes, median/mean HbA1c, diagnostic test and year of study conduction. All analyses will be performed with *R* statistical software 3.3.2. and p-values < 0.05 will be used as the threshold for statistical significance. Assessment of meta-bias(es) is not planned, as preliminary searches of registered protocols have indicated that it is unlikely that eligible epidemiological studies are prospectively registered. As this systematic review is not investigating the evidence underpinning an intervention, confidence in the cumulative evidence cannot be calculated or assessed using the Grading of Recommendations Assessment, Development and Evaluation. The strength of the body of evidence will be examined through the assessment of risk of bias within included studies.

For peer review only

## Discussion

Diabetic retinopathy is, in the early stages, an important marker of disease control, and in the later stages, a preventable cause of visual impairment and blindness. The burden of childhood diabetes is increasing, but there is a paucity of synthesised data on the prevalence and natural history of childhood diabetic retinopathy. This systematic review, using a clinically taxonomy to calculate the prevalence of diabetic retinopathy, will provide currently unavailable prevalence estimates to inform screening policy and practice for children and young people living with diabetes.

Ethics and dissemination

Ethical approval is not needed as this study will use only anonymised published data. We will report the findings of this systematic review in a peer-reviewed journal, and share it with the relevant professionals including health service planners through our DECS collaborative network.

For peer review only

## References

1. Liew G, Michaelides M, Bunce C. A comparison of the causes of blindness certifications in England and Wales in working age adults (16-64 years), 1999-2000 with 2009-2010. *BMJ Open.* 2014;4(2):e004015.
2. Haines L, Wan KC, Lynn R, Barrett TG, Shield JP. Rising incidence of type 2 diabetes in children in the U.K. *Diabetes Care.* 2007;30(5):1097-101.
3. Patterson CC, Dahlquist GG, Gyürüs E, Green A, Soltész G; EURODIAB Study Group. Incidence trends for childhood type 1 diabetes in Europe during 1989-2003 and predicted new cases 2005-20: a multicentre prospective registration study. *Lancet.* 2009;373(9680):2027-33. doi: 10.1016/S0140-6736(09)60568-7.
4. Dabelea D, Mayer-Davis EJ, Saydah S, Imperatore G, Linder B, Divers J, Bell R, Badaru A, Talton JW, Crume T, Liese AD, Merchant AT, Lawrence JM, Reynolds K, Dolan L, Liu LL, Hamman RF; SEARCH for Diabetes in Youth Study. Prevalence of type 1 and type 2 diabetes among children and adolescents from 2001 to 2009. *JAMA*. 2014;311(17):1778-86. doi: 10.1001/jama.2014.3201.
5. American Diabetes Association. Microvascular complications and foot care. Sec. 9. In Standards of Medical Care in Diabetes—2016. *Diabetes Care.* 2016;39(Suppl.1):S72-80.
6. Donaghue KC, Wadwa RP, Dimeglio LA, Wong TY, Chiarelli F, Marcovecchio ML, Salem M, Raza J, Hofman PL, Craig ME; International Society for Pediatric and Adolescent Diabetes. ISPAD Clinical Practice Consensus Guidelines 2014. Microvascular and macrovascular complications in children and adolescents. *Pediatr Diabetes.* 2014;15(Suppl 20):257-69.
7. National Institute for Health and Care Excellence (NICE). Diabetes (type 1 and type 2) in children and young people: diagnosis and management. NICE guidelines [NG18] August 2015. [Accessed March 2017]. Available from: https://www.nice.org.uk/guidance/ng18
8. Beauchamp G, Boyle CT, Tamborlane WV, Miller KM, Libman IM, Haller MJ, Beck RW; T1D Exchange Clinic Network. Treatable Diabetic Retinopathy Is Extremely Rare Among Pediatric T1D Exchange Clinic Registry Participants. *Diabetes Care*. 2016;39(12):e218-e219.
9. Geloneck MM, Forbes BJ, Shaffer J, Ying GS, Binenbaum G. Ocular Complications in Children with Diabetes Mellitus. *Ophthalmology.* 2015;122(12):2457-64. doi: 10.1016/j.ophtha.2015.07.010.
10. Hamid A, Wharton HM, Mills A, Gibson JM, Clarke M, Dodson PM. Diagnosis of retinopathy in children younger than 12 years of age: implications for the diabetic eye screening guidelines in the UK. *Eye (Lond).* 2016;30(7):949-51. doi: 10.1038/eye.2016.59.
11. Wang SY, Andrews CA, Herman WH, Gardner TW, Stein JD. Incidence and Risk Factors for Developing Diabetic Retinopathy among Youths with Type 1 or Type 2 Diabetes throughout the United States. *Ophthalmology.* 2017;124(4):424-430. doi: 10.1016/j.ophtha.2016.10.031.
12. Dabelea D, Stafford JM, Mayer-Davis EJ, D'Agostino R Jr, Dolan L, Imperatore G, Linder B, Lawrence JM, Marcovina SM, Mottl AK, Black MH, Pop-Busui R, Saydah S, Hamman RF, Pihoker C; SEARCH for Diabetes in Youth Research Group. Association of Type 1 Diabetes vs Type 2 Diabetes Diagnosed During Childhood and Adolescence With Complications During Teenage Years and Young Adulthood. *JAMA.* 2017;317(8):825-835. doi: 10.1001/jama.2017.0686.

For peer review only

13. Dinnes J, Deeks J, Kirby J, Roderick P. A methodological review of how heterogeneity has been examined in systematic reviews of diagnostic test accuracy. *Health Technol Assess*. 2005;9(12):1-113, iii.
14. Ibanez-Bruron MC, Solebo AL, Rahi JS. Complications of Diabetes Diagnosed in Children and Adolescents. *JAMA.* 2017;317(24):2553. doi:10.1001/jama.2017.6231.
15. Lueder GT, Silverstein J; American Academy of Pediatrics Section on Ophthalmology and Section on Endocrinology. Screening for retinopathy in the pediatric patient with type 1 diabetes mellitus. *Pediatrics*. 2005;116(1):270-3.
16. Thomas RL, Harvey JN, Owens DR. When should screening for diabetic retinopathy begin for children with type 1 diabetes? *Expert Review of Endocrinology & Metabolism.* 2016;11(2): 97-102. doi: 10.1586/17446651.2016.1145542.
17. Maguire A, Chan A, Cusumano J, Hing S, Craig M, Silink M, Howard N, Donaghue K. The case for biennial retinopathy screening in children and adolescents. *Diabetes Care.* 2005;28(3):509-13.
18. Moher D, Shamseer L, Clarke M, Ghersi D, Liberati A, Petticrew M, Shekelle P, Stewart LA. Preferred Reporting Items for Systematic Review and Meta-Analysis Protocols (PRISMA-P) 2015 statement. *Syst Rev*. 2015;4(1):1. doi: 10.1186/2046-4053-4-1
19. Klein R, Klein BE, Moss SE, Cruickshanks KJ. Relationship of hyperglycemia to the long-term incidence and progression of diabetic retinopathy. *Arch Intern Med.* 1994;154(19):2169-78.
20. Zhang X, Saaddine JB, Chou CF, Cotch MF, Cheng YJ, Geiss LS, Gregg EW, Albright AL, Klein BE, Klein R. Prevalence of diabetic retinopathy in the United States, 2005-2008. *JAMA.* 2010;304(6):649-56. doi: 10.1001/jama.2010.1111.
21. Hoy D, Brooks P, Woolf A, Blyth F, March L, Bain C, Baker P, Smith E, Buchbinder R Assessing risk of bias in prevalence studies: modification of an existing tool and evidence of interrater agreement. *J Clin Epidemiol.* 2012;65(9):934-9. doi: 10.1016/j.jclinepi.2011.11.014.
22. Higgins JP, Thompson SG. Quantifying heterogeneity in a meta-analysis. *Stat Med*. 2002;21(11):1539-58.

For peer review only

**Authors' contributions**

JSR is the guarantor. MIB designed the study and drafted the protocol. ALS and JSR critically revised and provided feedback on the protocol. All authors read and approved the final version of the manuscript.

**Funding**

This work was funded by the Ulverscroft Foundation, the National Institute for Health Research (NIHR) Biomedical Research Centre at University College London Institute of Child Health / Great Ormond Street Hospital NHS Foundation Trust, Diabetes Research & Wellness Foundation and the National Commission for Scientific and Technological Research in Chile (CONICYT). The funding organisations had no role in the design or conduct of this research.

**Competing interests**

The authors declare that they have no competing interests.

**Patient consent:** None

For peer review only

## Tables

**Table 1.** Search strategy MEDLINE (OvidSP)

| Category | # | Keyword |
|---|---|---|
| Children and young people | 1. | exp Child/ |
| | 2. | exp Adolescent/ |
| | 3. | exp Pediatrics/ |
| | 4. | child*.mp. |
| | 5. | teen*.mp. |
| | 6. | school age.mp. |
| | 7. | 1 /OR 6 |
| Diabetes | 8. | exp Diabetes Mellitus, Type 1/ |
| | 9. | exp Diabetes Mellitus, Type 2/ |
| | 10. | exp Diabetes Complications/ |
| | 11. | diabet*.mp. |
| | 12. | 8 OR/11 |
| Retinopathy | 13. | exp Eye Diseases/ |
| | 14. | exp Diabetic Retinopathy/ |
| | 15. | exp Retinal Neovascularization/ |
| | 16. | exp Macular Edema/ |
| | 17. | retinopath*.mp. |
| | 18. | maculopath*.mp. |
| | 19. | background retinopathy.mp. |
| | 20. | microaneurysm*.mp. |
| | 21. | 13 OR/20 |
| Prevalence | 22. | exp Epidemiology/ |
| | 23. | exp Prevalence/ |
| | 24. | exp Incidence/ |
| | 25. | exp Natural History/ |
| | 26. | frequenc*.mp. |
| | 27. | epidemiolog*.mp. |
| | 28. | prevalen*.mp. |
| | 29. | inciden*.mp. |
| | 30. | severity.mp. |
| | 31. | 22 OR/ 30 |
| Diagnostic tests | 32. | exp Photography/ |
| | 33. | exp Ophthalmoscopes/ |
| | 34. | exp Fluorescein Angiography/ |
| | 35. | exp Fundus Oculi/ |
| | 36. | exp Slit Lamp/ |
| | 37. | exp ophthalmoscopy/ |
| | 38. | Tomography, Optical Coherence/ |
| | 39. | Ultrasonography/ |
| | 40. | photo*.mp. |
| | 41. | ophthalmoscop* .mp. |
| | 42. | angiogram* .mp. |
| | 43. | biomicroscopy.mp. |
| | 44. | Optical coherence tomography.mp. |
| | 45. | ultrasonograph*.mp. |
| | 46. | fundus.mp. |
| | 47. | Slim Lamp.mp. |
| | 48. | fundoscop*.mp. |

For peer review only

| | |
|---|---|
| 49. | 32 OR/ 48 |
| 50. | 7 AND 12 AND 21 AND 31 AND 49 |
| 51. | limit 50 to yr="1995 -Current" |

For peer review only

**Table 2.** Inclusion and exclusion criteria.

| Category | Inclusion criteria | Exclusion criteria |
|---|---|---|
| Population | - Children and young people < 18 years old included in the sample.<br>- Children and young people from general population. | - Median/mean sample age > 25 years.<br>- Participants >30 years old included.<br>- Studies conducted in selected populations (e.g. dialysis, post-transplanted). |
| Exposure | - Type 1 or 2 diabetes. | - Other types of diabetes (e.g. monogenic causes) |
| Outcome of interest | - Prevalence of diabetic retinopathy.<br>- Test used to assess diabetic retinopathy reported. | - Estimation of prevalence not possible with published data. |
| Article | - Published between January 01, 1995 and Oct 31, 2016. | - Duplicate reports (the most comprehensive version will be included) |

For peer review only

For peer review only

**Additional file 1: PRISMA-P checklist**

**PRISMA-P (Preferred Reporting Items for Systematic review and Meta-Analysis Protocols) 2015 checklist: recommended items to address in a systematic review protocol***

| Section and topic | Item No | Checklist item | |
|---|---|---|---|
| **ADMINISTRATIVE INFORMATION** | | | |
| Title: | | | |
| Identification | 1a | Identify the report as a protocol of a systematic review | 1 |
| Update | 1b | If the protocol is for an update of a previous systematic review, identify as such | NA |
| Registration | 2 | If registered, provide the name of the registry (such as PROSPERO) and registration number | 3 |
| Authors: | | | |
| Contact | 3a | Provide name, institutional affiliation, e-mail address of all protocol authors; provide physical mailing address of corresponding author | 1 |
| Contributions | 3b | Describe contributions of protocol authors and identify the guarantor of the review | 12 |
| Amendments | 4 | If the protocol represents an amendment of a previously completed or published protocol, identify as such and list changes; otherwise, state plan for documenting important protocol amendments | NA |
| Support: | | | |
| Sources | 5a | Indicate sources of financial or other support for the review | 12 |
| Sponsor | 5b | Provide name for the review funder and/or sponsor | 12 |
| Role of sponsor or funder | 5c | Describe roles of funder(s), sponsor(s), and/or institution(s), if any, in developing the protocol | 12 |
| **INTRODUCTION** | | | |
| Rationale | 6 | Describe the rationale for the review in the context of what is already known | 4 |

For peer review only

| | | | |
|---|---|---|---|
| Objectives | 7 | Provide an explicit statement of the question(s) the review will address with reference to participants, interventions, comparators, and outcomes (PICO) | 4-5 |
| **METHODS** | | | |
| Eligibility criteria | 8 | Specify the study characteristics (such as PICO, study design, setting, time frame) and report characteristics (such as years considered, language, publication status) to be used as criteria for eligibility for the review | 6,15 |
| Information sources | 9 | Describe all intended information sources (such as electronic databases, contact with study authors, trial registers or other grey literature sources) with planned dates of coverage | 5 |
| Search strategy | 10 | Present draft of search strategy to be used for at least one electronic database, including planned limits, such that it could be repeated | 13-14 |
| Study records: | | | |
| Data management | 11a | Describe the mechanism(s) that will be used to manage records and data throughout the review | 7 |
| Selection process | 11b | State the process that will be used for selecting studies (such as two independent reviewers) through each phase of the review (that is, screening, eligibility and inclusion in meta-analysis) | 7 |
| Data collection process | 11c | Describe planned method of extracting data from reports (such as piloting forms, done independently, in duplicate), any processes for obtaining and confirming data from investigators | 7 |
| Data items | 12 | List and define all variables for which data will be sought (such as PICO items, funding sources), any pre-planned data assumptions and simplifications | 7 |
| Outcomes and prioritization | 13 | List and define all outcomes for which data will be sought, including prioritization of main and additional outcomes, with rationale | 6 |
| Risk of bias in individual studies | 14 | Describe anticipated methods for assessing risk of bias of individual studies, including whether this will be done at the outcome or study level, or both; state how this information will be used in data synthesis | 7,8, additional file. |
| Data synthesis | 15a | Describe criteria under which study data will be quantitatively synthesised | 8 |
| | 15b | If data are appropriate for quantitative synthesis, describe planned summary measures, methods of handling data and methods of combining data from studies, including any planned exploration of consistency (such as $I^2$, Kendall's τ) | 8 |
| | 15c | Describe any proposed additional analyses (such as sensitivity or subgroup analyses, meta-regression) | 8 |
| | 15d | If quantitative synthesis is not appropriate, describe the type of summary planned | 8 |
| Meta-bias(es) | 16 | Specify any planned assessment of meta-bias(es) (such as publication bias across studies, selective reporting | 8 |

For peer review only

| | | | |
|---|---|---|---|
| | | within studies) | |
| Confidence in cumulative evidence | 17 | Describe how the strength of the body of evidence will be assessed (such as GRADE) | 8 |

*** It is strongly recommended that this checklist be read in conjunction with the PRISMA-P Explanation and Elaboration (cite when available) for important clarification on the items. Amendments to a review protocol should be tracked and dated. The copyright for PRISMA-P (including checklist) is held by the PRISMA-P Group and is distributed under a Creative Commons Attribution Licence 4.0.**

*From: Shamseer L, Moher D, Clarke M, Ghersi D, Liberati A, Petticrew M, Shekelle P, Stewart L, PRISMA-P Group. Preferred reporting items for systematic review and meta-analysis protocols (PRISMA-P) 2015: elaboration and explanation. BMJ. 2015 Jan 2;349(jan02 1):g7647.*

For peer review only

**Additional file 2: Quality assessment**

__External validity__

1. **Was the sampling frame a true or close representation of the target population?**

Yes: All patients registered in paediatric diabetes units or clinics, i.e. general population of children and young people living with diabetes.

No: Participants selected only among screening attenders, voluntary participants of diabetes summer camps or other events.

**2. Are the study subjects described in detail regarding?**

Yes: Age or diabetes duration was reported along with a measure of diabetes control and the year(s) when the study was conducted.

No: One or more of the previous details was not described.

**3. Was some form of consecutive, random selection used to select the sample, OR was a census undertaken?**

Yes: Yes.

No: No or unclear.

**4. Was the likelihood of nonresponse bias minimal?**

Yes: Response >50%.

No: Unclear, not reported, or less 50%.

__Internal validity__

**5. Were data collected directly from the subjects (as opposed to a proxy)?**

Yes: Cross-sectional and prospective studies, retrospective analysis of prospective databases e.g. diabetic retinopathy screening registers.

No: Retrospective analysis of medical records.

**6. Was an acceptable case definition used in the study?**

Yes: Internationally acceptable (DCCT compliant) taxonomy of diabetic retinopathy was used.

No: No clear definition of diabetic retinopathy, e.g. *retinal changes,* or the severity of cases was not described.

**7. Was the study instrument that measured the parameter of interest shown to have validity and reliability?**

Yes: Retinal photographs or fluorescein angiogram.

No: Fundoscopy or ophthalmoscopy alone.

For peer review only

**8. Was the same mode of data collection used for all subjects?**

Yes: all participants received the same diagnostic test including screening tests.

No: Triage tests were used or unclear.

**9. Was the length of the shortest prevalence period for the parameter of interest appropriate?**

Yes: all studies were considered adequate. This is a crucial question in the prevalence of diabetic retinopathy in children and young people.

**10. Were the numerator(s) and denominator(s) for the parameter of interest appropriate and confidence interval given?**

Yes: Confidence interval given.

No: Confidence interval not given.

**11. Was the sample size equal or greater than 200?**

Yes.

No.

For peer review only

# Prevalence of diabetic retinopathy in children and young people living with diabetes: protocol for a systematic review.

| | |
|---|---|
| Journal: | *BMJ Open* |
| Manuscript ID | bmjopen-2017-018578.R1 |
| Article Type: | Protocol |
| Date Submitted by the Author: | 12-Oct-2017 |
| Complete List of Authors: | Ibanez-Bruron, Maria; UCL, GOS Institute of Child Health; UCL, Ulverscroft Vision Research Group<br>Solebo, AL; UCL, GOS Institute of Child Health; UCL, Ulverscroft Vision Research Group<br>Cumberland, Phillippa ; UCL, GOS Institute of Child Health; UCL, Ulverscroft Vision Research Group<br>Rahi, Jugnoo; UCL, GOS Institute of Child Health; UCL, Ulverscroft Vision Research Group |
| <b>Primary Subject Heading</b>: | Ophthalmology |
| Secondary Subject Heading: | Paediatrics, Diabetes and endocrinology, Epidemiology, Evidence based practice |
| Keywords: | Diabetic retinopathy < DIABETES & ENDOCRINOLOGY, EPIDEMIOLOGY, Paediatric ophthalmology < OPHTHALMOLOGY, PREVENTIVE MEDICINE |
| | |

SCHOLARONE™
Manuscripts

For peer review only

## Title page

**Title:**

Prevalence of diabetic retinopathy in children and young people living with diabetes: protocol for a systematic review.

**Authors**:
Maria C. Ibanez-Bruron MD; Ameenat L. Solebo FRCOphth; Phillippa M. Cumberland MSc; Jugnoo S. Rahi FRCOphth.

**Institutional address:**
GOS Institute of Child Health UCL, 30 Guildford Street, London, WC1N 1EH.

**Email addresses:**
m.bruron@ucl.ac.uk; a.solebo@ucl.ac.uk; p.cumberland@ucl.ac.uk; j.rahi@ucl.ac.uk

**Affiliations of authors:**
GOS Institute of Child Health, University College London, London, UK (MCI-B, ALS, PMC, JSR); Ulverscroft Vision Research Group, London, UK (MCI-B, ALS, PMC, JSR); Great Ormond Street Hospital for Children NHS Foundation Trust, London, UK (ALS, JSR); Moorfields Eye Hospital NHS Foundation Trust, London, UK (ALS, JSR); Institute of Ophthalmology, University College London, London, UK (ALS, JSR).

**Corresponding author:**
Professor Jugnoo Sangeeta Rahi FRCOphth.
GOS Institute of Child Health UCL, 30 Guildford Street, London, WC1N 1EH.
Tel: 020 7905 2250; email: j.rahi@ucl.ac.uk

For peer review only

## Abstract

**Introduction:** The frequency of diabetes mellitus in childhood is increasing. Thus, more children and young people are at risk of developing diabetic retinopathy and diabetes related visual impairment. However, there is no consensus on optimal screening strategies for the paediatric population reflecting the lack of clarity about the current burden of disease in this group. We aim to estimate the prevalence of diabetic retinopathy in children and young people living with types 1 or 2 diabetes, and to investigate potential sources of heterogeneity in this figure so as to inform screening strategies for this population.

**Methods and analysis:** PubMed and EMBASE will be searched from 1995 to 2016 using the OvidSP platform with no language restriction. Additionally, manual review of the references lists of included articles will be conducted. Two investigators will independently screen titles and abstracts for potential eligibility. Studies which report prevalence of diabetic retinopathy amongst general populations of children and young people with types 1 or 2 diabetes will be included. Pooled prevalence estimates of diabetic retinopathy reported in studies with sample size greater than 200 participants will be calculated by random effect model. Forest plots will be used to summarize individual and pooled estimates of the prevalence. Heterogeneity between studies will be assessed using the $I^2$ statistic and explored through meta-regressions and sub-group analyses if the necessary data are available.

**Ethics and dissemination:** Ethics approval is not required as this is a review of anonymised published data. We will report the findings of this systematic review in a peer-reviewed journal, and share it with the relevant professionals including health authorities through our DECS collaborative network.


For peer review only

**Systematic review registration:** PROSPERO (CRD42017067178).

**Strengths and limitations of this study**

- This formal systematic review will provide currently unavailable information on the prevalence of diabetic retinopathy in the paediatric population.
- Only studies reporting diagnostic methods and grades of diabetic retinopathy will be included to enhance precision.
- To increase the applicability of the findings, two severity cut-offs will be used to calculate prevalence estimates.
- As with all systematic reviews, the poor reporting of methods in primary studies might compromise the exploration of the heterogeneity.

For peer review only

## Background

Diabetic retinopathy is the most common preventable cause of visual impairment in young adults. [1] The rising prevalence of childhood type 1 and type 2 diabetes, [2-4] has led to a significant increase in the number of children and young people at risk of visual loss.

Microvascular retinal damage is a marker of general vascular health, indicative of a need for greater diabetic disease control. The more advanced, but still asymptomatic, stages of diabetic retinopathy require additional ophthalmic intervention to reduce the risk of irreversible visual loss. Screening for diabetic retinopathy in children and young people is recommended by many national population health programmes, with commencement of annual retinal examination determined either by age (typically age 10 – 12 years) or duration of disease (typically 2-5 years from diagnosis). [5-7]

Although it has been suggested that these recommendations need to be revised based on recent evidence on the burden of the disease in this population, there is no consensus on the direction of these modifications. [8-12] Variable estimates of the prevalence of diabetic retinopathy may be due in part to heterogeneity between study populations, diagnostic methods, or definitions of diabetic retinopathy used in the studies. [13-14] Consensus about screening strategies for the paediatric population required greater clarity in evidence base regarding the natural history and the burden of disease in this group. [15-17]

The aim of this systematic review is to estimate the prevalence of diabetic retinopathy in children and young people living with type 1 or type 2 diabetes, and to investigate potential sources of heterogeneity such as study sample characteristics

For peer review only

(e.g. age, diabetes type and duration, and glycaemic control), diagnostic methods and definition of diabetic retinopathy to inform screening strategies for the paediatric population.

**Methods/Design**

The systematic review will be performed in accordance with the Preferred Reporting Items for Systematic Reviews and Meta-Analyses (PRISMA) guidelines, with this protocol written according to the PRISMA-P checklist. [18] The PRISMA-P checklist can be found as Additional file 1. The study has been registered on PROSPERO (CRD42017067178).

**Data sources and searches**

The PubMed and EMBASE databases will be searched using the OvidSP platform. We will also manually review the references lists of identified articles. The search strategy (table 1) will include terms related to children or young people, diabetes, diabetic retinopathy, prevalence and diagnostic tests, and there will be no language restrictions. We will exclude studies published before January 1995 as older studies are likely to have been conducted prior to the publication of the Diabetes Control and Complications Trial (DCCT), which demonstrated decreased risk of diabetic retinopathy development or progression with tighter glycaemic control than that previously used in routine clinical practice, and may have led to significant changes in disease management and natural history. [19]

**Study eligibility criteria**

We will include studies which report prevalence estimates of diabetic retinopathy amongst general populations of children and young people with diabetes mellitus, or

For peer review only

studies from which the prevalence data may be derived (table 2). Eligible studies will be those which meet the following criteria:

- Participants

Individuals aged under 18 years living with types 1 or 2 diabetes. Studies with median/mean age of the sample greater than 25 years or which include participants older than 30 years will be excluded.

- Disease measure

Report of the clinical examination method used to determine diabetic retinopathy status, and description of either retinal findings or grade of retinopathy using any grading systems which can be correlated to retinopathy features seen at the ophthalmic examination. This will allow comparison between the different grading systems used in screening programmes and clinical practice. Included grading systems will be displayed in a supplementary table along with the systematic review's results.

Prevalence will be calculated using two cut-offs, *any* diabetic retinopathy and s*ight-threatening* diabetic retinopathy i.e. grades of retinopathy that require prompt ophthalmic management to reduce the risk of irreversible visual impairment. *Any* diabetic retinopathy will be defined as background retinopathy or more severe disease. *Sight-threatening* diabetic retinopathy [20] will be primarily defined as proliferative diabetic retinopathy or *clinically significant* maculopathy edema, i.e. retinal thickening at or within 500 μm of the center of the macula; and/or hard exudates at or within 500 μm of the center of the macula, if associated with thickening of the adjacent retina; and/or a zone or zones of retinal thickening one disc area in size, any part of which is within one disc

diameter of the center of the macula.[21] Additionally, a secondary analysis using a more inclusive definition of sight threatening diabetic retinopathy, i.e. including severe non-proliferative diabetic retinopathy, will be conducted using the available data.

Conference abstracts with sufficient data on disease measure to determine eligibility will be included.

**Study selection**

The search results will be extracted and managed using Microsoft Excel (Microsoft Corporation, Redmond, WA, USA). Two independent investigators (MIB, ALS) will screen titles and abstracts, and then apply the eligibility criteria to full-text articles. Disagreements will be resolved by consensus following discussion. Arbitration by a third author (JSR) will be sought whenever necessary.

**Data extraction**

Two investigators (MIB, ALS) will extract the data using a standardised form. The following data items will be extracted: first author, year of publication, design, country, year of study conduction, sample size, diabetes type, age at examination, diabetes duration, HbA1c, diagnostic test, diabetic retinopathy definition, number of any diabetic retinopathy cases, severity of diabetic retinopathy cases. If a study reports multiple prevalence estimates over time, the first prevalence estimate, i.e. the estimate which is reported with baseline sample clinical characteristics, will be selected as the most informative measure. Characteristics of children and young people with sight-threatening diabetic retinopathy will be also extracted. Authors will be contacted if data in the original publication is unclear.

For peer review only

## Assessment of risk of bias in included studies

The risk of bias will be assessed with an adapted version of the tool developed by Hoy and colleagues [22] (additional file 2). The sample will be considered representative of the target population (i.e. 'general' population) when all children and young people registered in a paediatric diabetes clinic, or living in a specified catchment area were potentially eligible for the study. Sources of information will be classified as appropriate where diabetic retinopathy status is assessed during the study (cross-sectional and prospective studies) or where prospective databases such as diabetic retinopathy screening registers are used.

## Data synthesis

A comprehensive table for summary of findings with narrative description of all eligible studies will be reported. However, only studies with a sample size equal or greater than 200 participants will be included in the meta-analysis. Small studies will be excluded to reduce variability due to imprecise estimates. Pooled prevalence estimates of any diabetic retinopathy and of sight-threatening diabetic retinopathy will be calculated by random effect model meta-analysis. Forest plots will be used to summarize individual and pooled estimates of the prevalence. Heterogeneity between studies will be assessed using the $I^2$ statistic (values of 25%, 50% and 75% will be considered low, medium and high heterogeneity, respectively) [23] and will be further explored through meta-regressions and sub-group analyses considering the following covariates: sample median/mean age, median/mean diabetes duration, type of diabetes, median/mean HbA1c, diagnostic test and year of study conduction. All analyses will be performed with *R* statistical software 3.3.2., and p-values < 0.05 will be used as the threshold for statistical significance. Assessment of meta-bias(es) is not planned, as preliminary searches of registered protocols have indicated that it

For peer review only

is unlikely that eligible epidemiological studies are prospectively registered. As this systematic review is not investigating the evidence underpinning an intervention, confidence in the cumulative evidence cannot be calculated or assessed using the Grading of Recommendations Assessment, Development and Evaluation. The strength of the body of evidence will be examined through the assessment of risk of bias within included studies.

**Discussion**

Diabetic retinopathy is, in the early stages, an important marker of disease control, and in the later stages, a preventable cause of visual impairment and blindness. The burden of childhood diabetes is increasing, but there is a paucity of synthesised data on the prevalence and natural history of childhood diabetic retinopathy. This systematic review, using a clinically impactful taxonomy to calculate the prevalence of diabetic retinopathy, will provide currently unavailable prevalence estimates to inform screening policy and practice for children and young people living with diabetes.

Ethics and dissemination

Ethical approval is not needed as this study will use only anonymised published data. We will report the findings of this systematic review in a peer-reviewed journal, and share it with the relevant professionals including health service planners through our DECS collaborative network.

For peer review only

## References


1. Liew G, Michaelides M, Bunce C. A comparison of the causes of blindness certifications in England and Wales in working age adults (16-64 years), 1999-2000 with 2009-2010. BMJ Open. 2014;4(2):e004015.
2. Haines L, Wan KC, Lynn R, Barrett TG, Shield JP. Rising incidence of type 2 diabetes in children in the U.K. Diabetes Care. 2007;30(5):1097-101.
3. Patterson CC, Dahlquist GG, Gyürüs E, Green A, Soltész G; EURODIAB Study Group. Incidence trends for childhood type 1 diabetes in Europe during 1989-2003 and predicted new cases 2005-20: a multicentre prospective registration study. Lancet. 2009;373(9680):2027-33.
4. Dabelea D, Mayer-Davis EJ, Saydah S, et al.; SEARCH for Diabetes in Youth Study. Prevalence of type 1 and type 2 diabetes among children and adolescents from 2001 to 2009. JAMA. 2014;311(17):1778-86.
5. American Diabetes Association. Microvascular complications and foot care. Sec. 9. In Standards of Medical Care in Diabetes—2016. Diabetes Care. 2016;39(Suppl.1):S72-80.
6. Donaghue KC, Wadwa RP, Dimeglio LA, et al.; International Society for Pediatric and Adolescent Diabetes. ISPAD Clinical Practice Consensus Guidelines 2014. Microvascular and macrovascular complications in children and adolescents. Pediatr Diabetes. 2014;15(Suppl 20):257-69.
7. National Institute for Health and Care Excellence (NICE). Diabetes (type 1 and type 2) in children and young people: diagnosis and management. NICE guidelines [NG18] August 2015. [Accessed October 2017]. Available from: https://www.nice.org.uk/guidance/ng18
8. Beauchamp G, Boyle CT, Tamborlane WV, et al.; T1D Exchange Clinic Network. Treatable Diabetic Retinopathy Is Extremely Rare Among Pediatric T1D Exchange Clinic Registry Participants. Diabetes Care. 2016;39(12):e218-e219.
9. Geloneck MM, Forbes BJ, Shaffer J, Ying GS, Binenbaum G. Ocular Complications in Children with Diabetes Mellitus. Ophthalmology. 2015;122(12):2457-64.
10. Hamid A, Wharton HM, Mills A, Gibson JM, Clarke M, Dodson PM. Diagnosis of retinopathy in children younger than 12 years of age: implications for the diabetic eye screening guidelines in the UK. Eye (Lond). 2016;30(7):949-51.
11. Wang SY, Andrews CA, Herman WH, Gardner TW, Stein JD. Incidence and Risk Factors for Developing Diabetic Retinopathy among Youths with Type 1 or Type 2 Diabetes throughout the United States. Ophthalmology. 2017;124(4):424-430.
12. Dabelea D, Stafford JM, Mayer-Davis EJ, et al.; SEARCH for Diabetes in Youth Research Group. Association of Type 1 Diabetes vs Type 2 Diabetes Diagnosed During Childhood and Adolescence With Complications During Teenage Years and Young Adulthood. JAMA. 2017;317(8):825-835.
13. Dinnes J, Deeks J, Kirby J, Roderick P. A methodological review of how heterogeneity has been examined in systematic reviews of diagnostic test accuracy. Health Technol Assess. 2005;9(12):1-113, iii.
14. Ibanez-Bruron MC, Solebo AL, Rahi JS. Complications of Diabetes Diagnosed in Children and Adolescents. JAMA. 2017;317(24):2553.
15. Lueder GT, Silverstein J; American Academy of Pediatrics Section on Ophthalmology and Section on Endocrinology. Screening for retinopathy in the pediatric patient with type 1 diabetes mellitus. Pediatrics. 2005;116(1):270-3.


For peer review only

16. Thomas RL, Harvey JN, Owens DR. When should screening for diabetic retinopathy begin for children with type 1 diabetes? Expert Review of Endocrinology & Metabolism. 2016;11(2): 97-102.
17. Maguire A, Chan A, Cusumano J, et al. The case for biennial retinopathy screening in children and adolescents. Diabetes Care. 2005;28(3):509-13.
18. Moher D, Shamseer L, Clarke M, et al. Preferred Reporting Items for Systematic Review and Meta-Analysis Protocols (PRISMA-P) 2015 statement. Syst Rev. 2015;4(1):1.
19. Klein R, Klein BE, Moss SE, Cruickshanks KJ. Relationship of hyperglycemia to the long-term incidence and progression of diabetic retinopathy. Arch Intern Med. 1994;154(19):2169-78.
20. Zhang X, Saaddine JB, Chou CF, et al. Prevalence of diabetic retinopathy in the United States, 2005-2008. JAMA. 2010;304(6):649-56.
21. Kinyoun J, Barton F, Fisher M, Hubbard L, Aiello L, Ferris F 3rd. Detection of diabetic macular edema. Ophthalmoscopy versus photography--Early Treatment Diabetic Retinopathy Study Report Number 5. The ETDRS Research Group. Ophthalmology. 1989;96(6):746-50.
22. Hoy D, Brooks P, Woolf A, et al. Assessing risk of bias in prevalence studies: modification of an existing tool and evidence of interrater agreement. J Clin Epidemiol. 2012;65(9):934-9.
23. Higgins JP, Thompson SG. Quantifying heterogeneity in a meta-analysis. Stat Med. 2002;21(11):1539-58.

For peer review only

### Authors' contributions

JSR is the guarantor. MIB designed the study and drafted the protocol. ALS, PMC and JSR critically revised and provided feedback on the protocol. MIB, ALS, PMC, and JSR read and approved the final version of the manuscript.

### Funding

This work was funded by the Ulverscroft Foundation, the National Institute for Health Research (NIHR) Biomedical Research Centre at University College London Institute of Child Health / Great Ormond Street Hospital NHS Foundation Trust, Diabetes Research & Wellness Foundation and the National Commission for Scientific and Technological Research in Chile (CONICYT). Ameenat Lola Solebo and Jugnoo Rahi are also funded by the National Institute for Health Research (NIHR) Moorfields Biomedical Research Centre. The funding organisations had no role in the design or conduct of this research.

### Competing interests

The authors declare that they have no competing interests.

**Patient consent:** None

For peer review only

## Tables

**Table 1.** Search strategy MEDLINE (OvidSP)

| Category | # | Keyword |
|---|---|---|
| Children and young people | 1. | exp Child/ |
| | 2. | exp Adolescent/ |
| | 3. | exp Pediatrics/ |
| | 4. | child*.mp. |
| | 5. | teen*.mp. |
| | 6. | school age.mp. |
| | 7. | 1 /OR 6 |
| Diabetes | 8. | exp Diabetes Mellitus, Type 1/ |
| | 9. | exp Diabetes Mellitus, Type 2/ |
| | 10. | exp Diabetes Complications/ |
| | 11. | diabet*.mp. |
| | 12. | 8 OR/11 |
| Retinopathy | 13. | exp Eye Diseases/ |
| | 14. | exp Diabetic Retinopathy/ |
| | 15. | exp Retinal Neovascularization/ |
| | 16. | exp Macular Edema/ |
| | 17. | retinopath*.mp. |
| | 18. | maculopath*.mp. |
| | 19. | background retinopathy.mp. |
| | 20. | microaneurysm*.mp. |
| | 21. | 13 OR/20 |
| Prevalence | 22. | exp Epidemiology/ |
| | 23. | exp Prevalence/ |
| | 24. | exp Incidence/ |
| | 25. | exp Natural History/ |
| | 26. | frequenc*.mp. |
| | 27. | epidemiolog*.mp. |
| | 28. | prevalen*.mp. |
| | 29. | inciden*.mp. |
| | 30. | severity.mp. |
| | 31. | 22 OR/ 30 |
| Diagnostic tests | 32. | exp Photography/ |
| | 33. | exp Ophthalmoscopes/ |
| | 34. | exp Fluorescein Angiography/ |
| | 35. | exp Fundus Oculi/ |
| | 36. | exp Slit Lamp/ |
| | 37. | exp ophthalmoscopy/ |
| | 38. | Tomography, Optical Coherence/ |
| | 39. | Ultrasonography/ |
| | 40. | photo*.mp. |
| | 41. | ophthalmoscop* .mp. |
| | 42. | angiogram* .mp. |
| | 43. | biomicroscopy.mp. |
| | 44. | Optical coherence tomography.mp. |
| | 45. | ultrasonograph*.mp. |
| | 46. | fundus.mp. |
| | 47. | Slim Lamp.mp. |
| | 48. | fundoscop*.mp. |

For peer review only

| | |
|---|---|
| 49. | 32 OR/ 48 |
| 50. | 7 AND 12 AND 21 AND 31 AND 49 |
| 51. | limit 50 to yr="1995 -Current" |

For peer review only

**Table 2.** Inclusion and exclusion criteria.

| **Category** | **Inclusion criteria** | **Exclusion criteria** |
| --- | --- | --- |
| Population | - Children and young people < 18 years old included in the sample.<br>- Children and young people from general population. | - Median/mean sample age > 25 years.<br>- Participants >30 years old included.<br>- Studies conducted in selected populations (e.g. dialysis, post-transplanted). |
| Exposure | - Type 1 or 2 diabetes. | - Other types of diabetes (e.g. monogenic causes) |
| Outcome of interest | - Prevalence of diabetic retinopathy.<br>- Test used to assess diabetic retinopathy reported. | - Estimation of prevalence not possible with published data. |
| Article | - Published between January 01, 1995 and Oct 31, 2016. | - Duplicate reports (the most comprehensive version will be included) |

For peer review only

**Additional file 1: PRISMA-P checklist**

**PRISMA-P (Preferred Reporting Items for Systematic review and Meta-Analysis Protocols) 2015 checklist: recommended items to address in a systematic review protocol***

| Section and topic | Item No | Checklist item | |
|---|---|---|---|
| **ADMINISTRATIVE INFORMATION** | | | |
| Title: | | | |
| Identification | 1a | Identify the report as a protocol of a systematic review | 1 |
| Update | 1b | If the protocol is for an update of a previous systematic review, identify as such | NA |
| Registration | 2 | If registered, provide the name of the registry (such as PROSPERO) and registration number | 3 |
| Authors: | | | |
| Contact | 3a | Provide name, institutional affiliation, e-mail address of all protocol authors; provide physical mailing address of corresponding author | 1 |
| Contributions | 3b | Describe contributions of protocol authors and identify the guarantor of the review | 12 |
| Amendments | 4 | If the protocol represents an amendment of a previously completed or published protocol, identify as such and list changes; otherwise, state plan for documenting important protocol amendments | NA |
| Support: | | | |
| Sources | 5a | Indicate sources of financial or other support for the review | 12 |
| Sponsor | 5b | Provide name for the review funder and/or sponsor | 12 |
| Role of sponsor or funder | 5c | Describe roles of funder(s), sponsor(s), and/or institution(s), if any, in developing the protocol | 12 |
| **INTRODUCTION** | | | |
| Rationale | 6 | Describe the rationale for the review in the context of what is already known | 4 |

For peer review only

| | | | |
|---|---|---|---|
| Objectives | 7 | Provide an explicit statement of the question(s) the review will address with reference to participants, interventions, comparators, and outcomes (PICO) | 4-5 |
| **METHODS** | | | |
| Eligibility criteria | 8 | Specify the study characteristics (such as PICO, study design, setting, time frame) and report characteristics (such as years considered, language, publication status) to be used as criteria for eligibility for the review | 6,15 |
| Information sources | 9 | Describe all intended information sources (such as electronic databases, contact with study authors, trial registers or other grey literature sources) with planned dates of coverage | 5 |
| Search strategy | 10 | Present draft of search strategy to be used for at least one electronic database, including planned limits, such that it could be repeated | 13-14 |
| Study records: | | | |
| Data management | 11a | Describe the mechanism(s) that will be used to manage records and data throughout the review | 7 |
| Selection process | 11b | State the process that will be used for selecting studies (such as two independent reviewers) through each phase of the review (that is, screening, eligibility and inclusion in meta-analysis) | 7 |
| Data collection process | 11c | Describe planned method of extracting data from reports (such as piloting forms, done independently, in duplicate), any processes for obtaining and confirming data from investigators | 7 |
| Data items | 12 | List and define all variables for which data will be sought (such as PICO items, funding sources), any pre-planned data assumptions and simplifications | 7 |
| Outcomes and prioritization | 13 | List and define all outcomes for which data will be sought, including prioritization of main and additional outcomes, with rationale | 6 |
| Risk of bias in individual studies | 14 | Describe anticipated methods for assessing risk of bias of individual studies, including whether this will be done at the outcome or study level, or both; state how this information will be used in data synthesis | 7,8, additional file. |
| Data synthesis | 15a | Describe criteria under which study data will be quantitatively synthesised | 8 |
| | 15b | If data are appropriate for quantitative synthesis, describe planned summary measures, methods of handling data and methods of combining data from studies, including any planned exploration of consistency (such as $I^2$, Kendall's τ) | 8 |
| | 15c | Describe any proposed additional analyses (such as sensitivity or subgroup analyses, meta-regression) | 8 |
| | 15d | If quantitative synthesis is not appropriate, describe the type of summary planned | 8 |
| Meta-bias(es) | 16 | Specify any planned assessment of meta-bias(es) (such as publication bias across studies, selective reporting | 8 |

For peer review only

| | | | |
|---|---|---|---|
| | | within studies) | |
| Confidence in cumulative evidence | 17 | Describe how the strength of the body of evidence will be assessed (such as GRADE) | 8 |

*** It is strongly recommended that this checklist be read in conjunction with the PRISMA-P Explanation and Elaboration (cite when available) for important clarification on the items. Amendments to a review protocol should be tracked and dated. The copyright for PRISMA-P (including checklist) is held by the PRISMA-P Group and is distributed under a Creative Commons Attribution Licence 4.0.**

*From: Shamseer L, Moher D, Clarke M, Ghersi D, Liberati A, Petticrew M, Shekelle P, Stewart L, PRISMA-P Group. Preferred reporting items for systematic review and meta-analysis protocols (PRISMA-P) 2015: elaboration and explanation. BMJ. 2015 Jan 2;349(jan02 1):g7647.*

For peer review only

**Additional file 2: Quality assessment**

**External validity**

1. **Was the sampling frame a true or close representation of the target population?**

Yes: All patients registered in paediatric diabetes units or clinics, i.e. general population of children and young people living with diabetes.

No: Participants selected only among screening attenders, voluntary participants of diabetes summer camps or other events.

**2. Are the study subjects described in detail regarding?**

Yes: Age or diabetes duration was reported along with a measure of diabetes control and the year(s) when the study was conducted.

No: One or more of the previous details was not described.

**3. Was some form of consecutive, random selection used to select the sample, OR was a census undertaken?**

Yes: Yes.

No: No or unclear.

**4. Was the likelihood of nonresponse bias minimal?**

Yes: Response >50%.

No: Unclear, not reported, or less 50%.

**Internal validity**

**5. Were data collected directly from the subjects (as opposed to a proxy)?**

Yes: Cross-sectional and prospective studies, retrospective analysis of prospective databases e.g. diabetic retinopathy screening registers.

No: Retrospective analysis of medical records.

**6. Was an acceptable case definition used in the study?**

Yes: Internationally acceptable (DCCT compliant) taxonomy of diabetic retinopathy was used.

No: No clear definition of diabetic retinopathy, e.g. *retinal changes,* or the severity of cases was not described.

**7. Was the study instrument that measured the parameter of interest shown to have validity and reliability?**

Yes: Retinal photographs or fluorescein angiogram.

No: Fundoscopy or ophthalmoscopy alone.

For peer review only

**8. Was the same mode of data collection used for all subjects?**

Yes: all participants received the same diagnostic test including screening tests.

No: Triage tests were used or unclear.

**9. Was the length of the shortest prevalence period for the parameter of interest appropriate?**

Yes: all studies were considered adequate. This is a crucial question in the prevalence of diabetic retinopathy in children and young people.

**10. Were the numerator(s) and denominator(s) for the parameter of interest appropriate and confidence interval given?**

Yes: Confidence interval given.

No: Confidence interval not given.

**11. Was the sample size equal or greater than 200?**

Yes.

No.

For peer review only
